# Assessing affymetrix GeneChip microarray quality

**DOI:** 10.1186/1471-2105-12-137

**Published:** 2011-05-07

**Authors:** Matthew N McCall, Peter N Murakami, Margus Lukk, Wolfgang Huber, Rafael A Irizarry

**Affiliations:** 1 Department of Biostatistics and Computational Biology, University of Rochester Medical Center, 601 Elmwood Ave., Rochester, NY, USA; 2 Center for Epigenetics, Johns Hopkins School of Medicine, 855 N. Wolfe St., Baltimore, MD, USA; 3 EMBL-EBI Functional Genomics Group, Wellcome Trust Genome Campus, Hinxton, Cambridge, CB10 1SD, UK; 4 Cancer Research UK Cambridge Research Institute, Li Ka Shing Centre, Robinson Way, Cambridge, CB2 ORE, UK; 5 EMBL Genome Biology Unit, 69117 Heidelberg, Germany; 6 Department of Biostatistics, Johns Hopkins Bloomberg School of Public Health, 615 N. Wolfe St., Baltimore, MD, USA

## Abstract

**Background:**

Microarray technology has become a widely used tool in the biological sciences. Over the past decade, the number of users has grown exponentially, and with the number of applications and secondary data analyses rapidly increasing, we expect this rate to continue. Various initiatives such as the External RNA Control Consortium (ERCC) and the MicroArray Quality Control (MAQC) project have explored ways to provide standards for the technology. For microarrays to become generally accepted as a reliable technology, statistical methods for assessing quality will be an indispensable component; however, there remains a lack of consensus in both defining and measuring microarray quality.

**Results:**

We begin by providing a precise definition of microarray quality and reviewing existing Affymetrix GeneChip quality metrics in light of this definition. We show that the best-performing metrics require multiple arrays to be assessed simultaneously. While such *multi-array *quality metrics are adequate for bench science, as microarrays begin to be used in clinical settings, single-array quality metrics will be indispensable. To this end, we define a single-array version of one of the best multi-array quality metrics and show that this metric performs as well as the best multi-array metrics. We then use this new quality metric to assess the quality of microarry data available via the Gene Expression Omnibus (GEO) using more than 22,000 Affymetrix HGU133a and HGU133plus2 arrays from 809 studies.

**Conclusions:**

We find that approximately 10 percent of these publicly available arrays are of poor quality. Moreover, the quality of microarray measurements varies greatly from hybridization to hybridization, study to study, and lab to lab, with some experiments producing unusable data. Many of the concepts described here are applicable to other high-throughput technologies.

## Background

Microarray technology has become a widely used tool in the biological sciences. Over the past decade, the number of users has grown exponentially, and with the number of applications and secondary data analyses rapidly increasing, we expect this rate to continue. Various initiatives such as the External RNA Control Consortium (ERCC) [1] and the MicroArray Quality Control (MAQC) projects [2,3] have explored ways to provide standards for the technology. For microarrays to become generally accepted as a reliable technology, statistical methods for assessing quality will be an indispensable component; however, there remains a lack of consensus in both defining and measuring microarray quality.

Defining quality in the context of a microarray experiment is not an easy task. The American Society for Quality (ASQ) defines quality as a subjective term for which each person has his or her own definition. In technical usage, quality can have two meanings: a product or service free of deficiencies, or the characteristics of a product or service that bear on its ability to satisfy stated or implied needs [4]. Many other definitions of quality exist but a common theme of most is the dependence of quality on the needs of the consumer. So what do users of gene expression microarrays want? The most common applications appear to be: finding differentially expressed genes between two conditions, clustering genes or samples, and predicting sample types or outcomes.

In our attempt to measure quality we quantify the effect of removing bad quality data on the biological results reported in a publication, which we refer to as bottom-line results. One should note that bottom-line results depend on the application. Furthermore, various levels of the data can be considered for removal; we can consider removing: one data point from a feature on one array, all data points from a feature across all arrays, all data from an array hybridization, all data arising from an RNA sample, all data arising from an entire batch of arrays, all data arising from an entire experiment/study, or, in a cross-study meta-analysis [5,6], all data produced from a particular lab. Thus, defining quality in the context of microarray experiments is indeed a difficult task. To provide a useful review, in this paper we focus our attention on the removal of all data from a poor quality array hybridization and the subsequent improvement in bottom-line results. Because most results from microarray studies combine data from various hybridizations, even one bad array can easily taint final results.

Recently, two factors have greatly increased the demand for reliable assessment of microarray quality: microarrays are beginning to be used in clinical settings to aid in diagnosis [7] and researchers are conducting meta-analyses and developing bioinformatic tools to mine the plethora of microarray data made publicly available through GEO and ArrayExpress [8-12]. In the former case, it is crucial to know whether the data being used to guide patient care is of usable quality. In the latter case, poor quality arrays might taint the results of a large meta-analysis or cause a bioinformatic tool to provide erroneous information.

In this paper, we focus on Affymetrix GeneChip microarrays, but many of the methods and recommendations presented can be extended to other microarray platforms and other high-throughput technologies for which there exists enough publicly available data. In the Methods Section, we begin by revisiting a widely used statistical model and discussing its implications regarding array quality. We then propose a formal definition of array quality and use this definition to assess the performance of current quality metrics. We find that single-array metrics typically perform poorly, while multi-array metrics perform well. While multi-array metrics are useful in traditional laboratory experiments, many modern uses of microarrays - such as clinical use, large meta-analyses, etc. - would benefit from single-array quality metrics. To address this, we propose a novel single-array quality metric based on one of the best multi-array quality metrics. We demonstrate that this single-array metric performs nearly identically to the multi-array metric on which it is based, except in a specific situation where the multi-array metric fails. Finally, because publicly available microarray data is often used to develop and test new algorithms and bioinformatic tools, we use our newly developed metric to assess the quality of publicly available Affymetrix microarray data.

## Methods

To better understand what we are measuring and what we actually observe, we use a relatively simple statistical model. This model has been proposed by various authors [13-15]:

Here *I*_*i*,*j *_represents the observed intensity for feature *i *for sample *j*. *K_j _*is an sample/array effect which accounts for the need for normalization, *θ*_*i*,*j *_represents a quantity proportional to the amount of RNA hybridized to the array (the quantity of interest), *ϕ_i _*quantifies the probe effect, *ε *represents measurement error and *O*_*i*,*j *_represent the components of the intensity due to non-specific binding and optical noise. For simplicity, we assume we can correct for the background components, *O*_*i*,*j*_, and that *K_j_*, *θ*_*i*,*j*_, and *ϕ_i _*are not zero. Under these assumptions we can simplify to a linear additive model; this model is extensively used as part of the Robust Multi-array Analysis (RMA) preprocessing algorithm [16]:(1)

This parametrization reveals two important facts for quality assessment. First, a feature intensity being larger on one array when compared to another does not imply the level of expression is also larger because K may differ. Using the notation above we write *Y*_*i*1 _>*Y*_*i*,2 _does not imply *θ*_*i*,1 _>*θ*_*i*,2_. Veterans of microarray data analysis know this very well and always perform normalization before making direct comparisons. Second, two feature intensities on the same array are not comparable because of the probe effect *ϕ_i_*. In other words, *Y*_1,*j *_>*Y*_2,*j *_does not imply *θ*_1,*j *_>*θ*_2,*j*_. This fact, although not explicitly explained in most papers, is the principal reason why most publications using microarray experiments base findings on relative or differential expression. Using the notation above and assuming we have normalized and removed the K, we can write:

where *δ_i _*is measurement error. In this case the probe-effect cancels out and the observed log ratio is a useful estimate of the true log ratio of expression levels.

### Quantifying Quality

We start by defining some notation. Let *A *= *A*_1_, ..., *A_N _*represent the data from *N *arrays. Denote with *f *the data manipulations that are performed on *A *to produce a set of results represented by *R*, i.e. let *f(A) *= *R*. Let *Q *represent a quantification of the accuracy and precision of *R*. We define a successful quality assessment procedure as one that prompts us to ignore data from array *j*, that is Δ*_j _*= *Q*(*A_-j_*) - *Q*(*A*) > 0. Here *A_-j _*represents the data set with the data from array *j *excluded and Δ*_j _*the improvement from removing the *j*th array. A specific example of the above notation is the following: *A *represents the data from 8 arrays (4 experimental samples compared to 4 control samples), *f *represents the action of computing the t-statistic for each gene and from this value computing an FDR q-value, *R *is the list of genes for which *q *< 0.05, and *Q *is the percentage of true and false positives on our list. Notice that removing an array of bad quality can result in improved accuracy and precision, but removing a good quality array can worsen the results because we lose power by considering less data. It is important to keep in mind that overzealous quality metrics can actually worsen results.

In general, *Q *is not computable. If we had a way to know true and false positives we would not need to run the experiment. However, for the purpose of assessing quality metrics, we need experiments with enough a-priori knowledge that we can define *Q*. It is very important to note that *Q *must be defined prior to observing *R*, e.g. it is not appropriate to define true positives based on the q-values obtained from *R*.

### Review of Existing Quality Assessments

Various summary statistics or quality metrics have been suggested for Affymetrix GeneChip arrays. Affymetrix's software offers 8 quality metrics; Bolstad et al. proposed two additional metrics [17]. A description of these methods follows.

#### Affymetrix Quality Metrics

Affymetrix provides various quality metrics as part of their MAS5.0 analysis software. Of these, the three most commonly used metrics are: average background, scale factor, and percent present. Other metrics provided by Affymetrix assess the quality of the RNA hybridized to the array rather than array quality itself. Average background is computed as the 2nd percentile of the feature intensities in a given region of the array. It is intended to measure optical background. Affymetrix considers average background values between 20 and 100 as typical for a good quality array. The scale factor is the median feature intensity on an array. Affymetrix normalizes arrays by scaling them based on these values. Within an experiment, arrays are expected to have scale factors within 3-fold of each other; arrays whose scale factors are outside this range are considered to have poor quality. The percent present is the percentage of genes called present by Affymetrix's detection algorithm [18]. These percentages should be similar between replicate samples, and arrays with extremely low values should be considered poor quality. We refer the reader to [18] for a more detailed description of these metrics.

#### Multi-array Quality Metrics

The first quality metric proposed by Bolstad et al. [17] is the relative log expression (RLE). These values are calculated by subtracting the median gene expression estimate across arrays from each gene expression estimate, . Therefore, the RLE for gene *i *on array *j *is:

For a given array, a median RLE not near zero indicates that the number of up-regulated genes does not approximately equal the number of down-regulated genes, and a large RLE IQR indicates that most genes are differentially expressed. If these indications are not biologically plausible, the array is likely of poor quality.

The second quality metric proposed by Bolstad et al. [17] is the normalized unscaled standard error (NUSE). For a given gene, *j*, the NUSE provides a measure of the precision of its expression estimate on a given array, *i*, relative to other arrays in the batch. Specifically, it is defined as:

Problematic arrays result in higher SEs than the median SE; therefore, arrays are suspected to be of poor quality if either the median NUSE is above one or they have a large IQR.

RLE and NUSE values can be displayed in boxplots and summarized with the median and interquartile range (IQR). Both RLE and NUSE values for any given array depend on the other arrays in the batch; therefore, values from different batches are not directly comparable. Also it is important to note that NUSE values depend on fitting Model 1, also known as the RMA model, but RLE values do not.

### Single-array Version of NUSE

A weakness of the approaches proposed by Affymetrix is that the probe-effect, described above, is not taken into consideration. A large proportion of the variation seen across feature intensities can be predicted by the probe-effect implying that the identification of outliers becomes easier when considering this effect. The alternative quality metrics proposed by Bolstad et al. do take the probe-effect into account; however, to estimate and adjust for probe-effects, the user is required to analyze multiple arrays simultaneously. Such *multi-array *methods borrow information across arrays which were hybridized under similar conditions allowing the probe-effects to be estimated. While they often provide far better performance, multi-array methods cannot be used in situations where a single array needs to be analyzed. An additional limitation of the methods proposed by Bolstad et al. is that they provide a relative measure of microarray quality not an absolute one. That is, RLE and NUSE values are only able to determine if an array's quality is better or worse than the typical array being analyzed in that experiment or batch. The methods proposed by Affymetrix are single-array and do not suffer from these limitations.

In order to obtain a single-array absolute measure of microarray quality, we propose a modification of the NUSE metric. We call this new metric a *Global NUSE *or *GNUSE *because the quality of an individual microarray is assessed relative to a balanced sample of all publicly available microarray data on a given platform. As such, it provides a *global *view of microarray quality. Specifically, we compute the median SE vector from a large biologically diverse data set and use this vector to normalize SE values from new arrays. We define a global normalized unscaled standard error (GNUSE) for a given gene, *j*, on array, *i*, as:

where *i *= 1, ..., *I *denotes all the arrays in the larger data set. In this paper *I *= 1, 000, as we used the same 1,000 samples used to create the reference distribution for the current implementation of the frozen Robust Multi-array Analysis (fRMA) preprocessing algorithm [19]. By preprocessing arrays with fRMA, the values for  are directly comparable across arrays and batches. However, it should be noted that the median SE vector is platform-specific.

Similar to NUSE values, GNUSE values can be displayed using boxplots and summarized using the median and IQR with a median GNUSE greater than one or a large IQR indicative of poor quality.

## Results and Discussion

### Assessment of Quality Metrics

We first evaluate the quality metrics proposed by Affymetrix and Bolstad et al. based on their ability to provide good bottom-line results for each of the 3 primary applications of gene expression microarrays - differential expression, clustering, and sample type prediction. We show that the metrics proposed by Bolstad et al. are often able to detect poor quality arrays while the Affymetrix metrics typically fail to do so. Because in the first three assessments the NUSE and GNUSE values are nearly identical, we omit the GNUSE. In the fourth example, we provide a situation where the GNUSE provides more informative results.

#### Differential Expression

**Figure 1:**
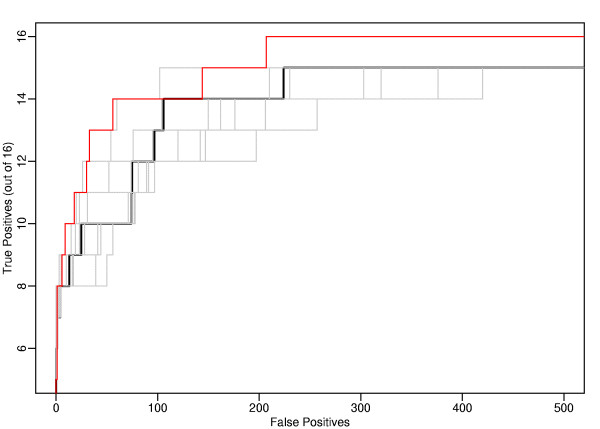
**Area under the ROC curve is increased by removing a poor quality array**. ROC curves for detection of the 16 spiked-in transcripts using all 8 arrays (black line) and with each array removed (gray lines). The red ROC curve corresponds to removing array 4. Removing array 4 results in the largest increase in the area under the ROC curve suggesting that array 4 may be of poor quality.

As our first example we use the data from Affymetrix's HGU95 spike-in study. In this experiment 16 transcripts were spiked in to background RNA in such a way that 59 arrays were replicated except for these 16 transcripts. We selected a subset of 8 arrays for which 2 sets of 4 arrays had identical spike-in concentrations - this is our array data *A*. We then performed a t-test comparing one group of 4 arrays to the other and obtained false discovery rates (FDRs) - this is the data manipulation *f*. For various FDR cut-offs we formed lists of candidate genes - our result *R*. A perfect list will only contain the 16 spiked-in transcripts, so we are able to calculate a quantification of accuracy and precision, *Q*. We repeated the analysis, this time removing each array one at a time. Based on each of these procedures, we plotted an ROC curve (Figure 1). For one particular array (in red), its removal noticeably improved results - a large Δ*_j_*. The q-values for the true positives further demonstrate the positive effect of removing this array (Table 1). Finally, a residual image shows the array in question has a very strong spatial effect (Additional file 1, Figure S1). Now the question is: which quality metric detects this array as problematic? The Affymetrix quality metrics suggest that the array has similar quality to others (Additional file 1, Figure S2); this is to be expected because Affymetrix presumably used their quality metrics to screen these arrays. However, the NUSE and RLE metrics correctly detect the array in questions as having poor quality.

**Table 1:** Q-values for True Positives

	Original	1	2	3	4	5	6	7	8
546_at	**<0.01**	<0.01	<0.01	<0.01	**<0.01**	<0.01	<0.01	<0.01	0.01
36311_at	**<0.01**	0.02	0.02	0.03	**<0.01**	<0.01	0.02	0.04	0.01
36889_at	**<0.01**	<0.01	0.03	0.03	**<0.01**	0.02	0.06	0.04	0.02
1091_at	**<0.01**	0.02	0.02	0.03	**<0.01**	0.01	0.02	0.02	0.01
39058_at	**<0.01**	0.02	0.02	0.03	**<0.01**	0.01	0.02	0.04	0.02
1024_at	**<0.01**	0.05	0.05	0.04	**<0.01**	0.02	0.1	0.1	0.02
37777_at	**0.02**	0.07	0.17	0.09	**<0.01**	0.1	0.31	0.42	0.04
684_at	**0.02**	0.08	0.13	0.09	**<0.01**	0.1	0.11	0.13	0.06
33818_at	**0.08**	0.11	0.24	0.49	**<0.01**	0.85	0.84	0.85	0.75
407_at	**0.39**	0.58	0.64	0.67	**0.14**	0.85	0.84	0.64	0.76
36202)_at	**0.52**	0.58	0.64	0.67	**<0.01**	0.85	0.84	0.85	0.76
1597_at	**0.75**	0.58	0.64	0.67	**0.73**	0.85	0.84	0.85	0.76
38734_at	**0.75**	0.58	0.64	0.67	**0.46**	0.85	0.84	0.85	0.76
36085_at	**0.75**	0.58	0.64	0.67	**0.46**	0.85	0.84	0.85	0.76
40322_at	**0.75**	0.58	0.64	0.67	**0.46**	0.85	0.84	0.85	0.76
1708_at	**0.75**	0.58	0.64	0.67	**0.61**	0.85	0.84	0.85	0.76

The q-values for each of the 16 spiked in probesets using all 8 arrays (Column 1) and with each of the 8 arrays removed (Columns 2-9). A q-value < 0.01 denotes a probeset correctly identified as differentially expressed. Removing the poor quality array (4) decreases the q-values, while removing the other good quality arrays increases the q-values.

#### Clustering

**Figure 2:**
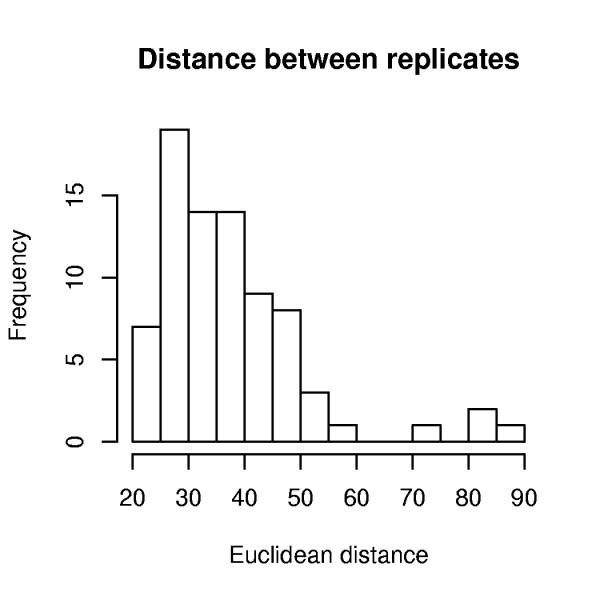
**Distances between replicate tissues**. Histogram of the Euclidean distance between the gene expression values for paired tissue samples. We expect these distances to be fairly small because we assume that, for the most part, the same genes are expressed in samples from the same tissue. In fact, this does appear to be the case - the typical distance between replicate samples is 30. However, one pair of replicate tissues (Cardiac Myocytes) has a distance larger than 90, suggesting one of the samples may be of poor quality.

For the second example we constructed a data set composed of two replicate arrays for 79 different tissues (*A*). We then pretended that we did not know the tissues and clustered all the samples using hierarchical clustering with Euclidean distance (Additional file 1, Figure S3) - this is *f*. Because we in fact know the tissues we can define *Q *as the average distance between replicates. The typical distance between replicates is 30 (Figure 2), but one pair of replicate tissues (Cardiac Myocytes) stands out as clearly problematic (distance >90). This suggests that one (or both) of the Cardiac Myocytes arrays has poor quality. The only metric that detects one of these arrays as problematic is the NUSE metric (Additional file 1, Figure S4). In these data we also noticed an additional sub-cluster (Additional file 1, Figure S3); these arrays are identified by the RLE metric as clearly problematic (Additional file 1, Figure S4). The NUSE and percent present metrics are also able to detect these arrays as being somewhat problematic. The poor quality Cardiac Myocytes array, detected by NUSE, has a strong spatial effect (Additional file 1, Figure S5). Residual images also show that the sub-cluster of arrays have different expression patterns in specific regions of the array (data not shown). This must be an artifact; a likely explanation is that Affymetrix organizes the probes in rows by sequence properties and sample preparation somehow favored certain probe sequences in the sub-cluster of arrays.

#### Prediction

The final test of a quality metric is whether removing poor quality arrays results in improved inference. To assess this, we considered predicting a clinical parameter, pathologic complete response (0 if residual disease; 1 otherwise), based on microarray data provided by MD Anderson to the MAQC-II project [3,20].

These data were divided into training and validation sets as part of the original study design. The only modification we made to these designations was to include 14 arrays that were flagged as poor quality by the original study participants. This resulted in 96 training samples and 51 test samples (*A*).

To investigate the effect of microarray quality on prediction, we fit a model to the training data using all 96 samples and made predictions on the test samples (*f*). We then removed the lowest quality array, refit the model, and made a new set of predictions. We repeated this procedure 50 times, each time removing one additional array and assessing the prediction by Matthews Correlation Coefficient (*Q*). For our prediction algorithm, we chose one of the most widely used algorithms - Prediction Analysis for Microarrays (PAM) [21]. This procedure was done for each of the quality metrics described above.

**Figure 3:**
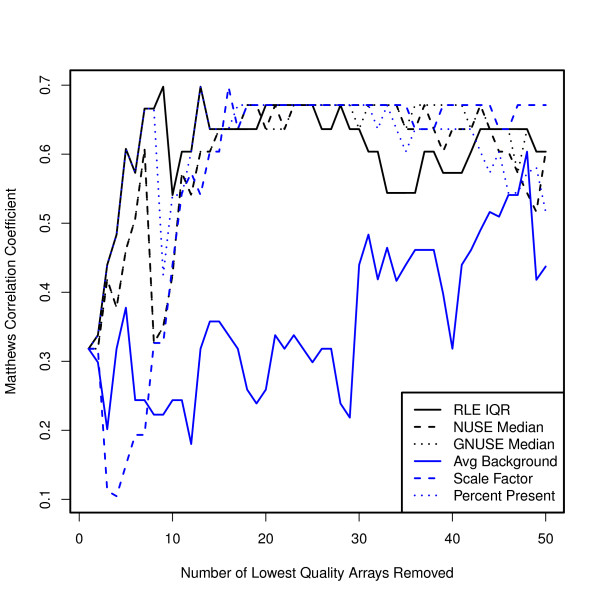
**Removing poor quality arrays improves prediction**. Plot of Matthews Correlation Coefficient for a clinical parameter, pathologic complete response, versus the number of lowest quality arrays removed for each quality metric. The prediction algorithm used was PAM. Prediction improved when removing the arrays with the poorest quality; however, some metrics did substantially better than others at detecting arrays that negatively affect prediction. RLE and Percent Present appeared to perform best, followed by NUSE and GNUSE. Average background showed no improvement when removing less than 30 arrays.

In general, we observed an improvement in prediction when removing the arrays with the poorest quality (Figure 3). However, some metrics did substantially better than others at detecting arrays that negatively affect prediction. In particular, the RLE and Percent Present appeared to perform best, followed by NUSE.

#### GNUSE vs. NUSE

Because most published experiments are composed of primarily good quality arrays, the GNUSE and NUSE values are often fairly similar. For example, we repeated the prediction analysis above using the GNUSE. Recall that out of the 96 training samples we expect most to be of good quality. The prediction improvement seen using GNUSE is nearly identical to that seen using NUSE (see Figure 3).

However, the GNUSE offers two advantages over the NUSE. First, GNUSE values can be obtained from a single array. Second, since the NUSE measures quality relative to other arrays in a batch, if most arrays in a batch are of poor quality, the denominator will be inflated and all arrays may appear to be of acceptable quality. The GNUSE is not susceptible to such errors because its denominator is computed based on a large fixed sample of arrays. This difference can be seen in boxplots of the NUSE and GNUSE values for a published data set comprised of a sizable number of poor quality arrays (Additional file 1, Figure S6). Notice that many of the arrays look acceptable based on the NUSE, whereas most appear to be of poor quality based on the GNUSE.

### Assessment of Publicly Available Data

Having developed a single-array quality metric (GNUSE) that performs at least as well as the best multi-array quality metrics, we turn our attention to assessing the quality of publicly available microarray data.

#### GEO Quality

**Figure 4:**
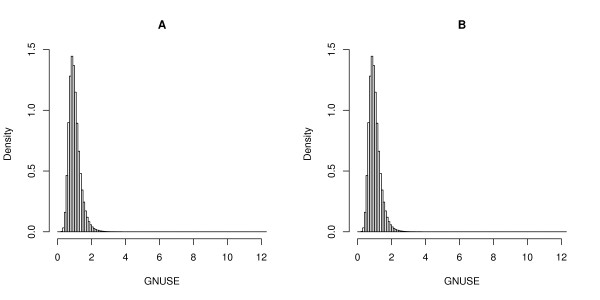
**Distribution of GNUSE values**. Histograms of GNUSE values from (A) 11299 HGU133a arrays from 338 studies and (B) 11029 HGU133plus2 arrays from 471 studies. Most GNUSE values are of acceptable quality (close to one), but the long right tail suggests some very poor quality probesets.

**Figure 5:**
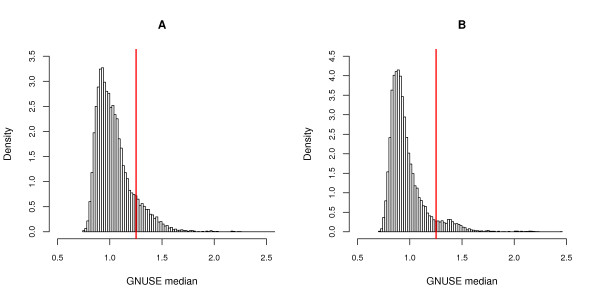
**Distribution of median GNUSE values**. Histograms of median GNUSE values from (A) 11299 HGU133a arrays or (B) 11029 HGU133plus2 arrays. The red vertical line represents the threshold of 1.25 - arrays with a median GNUSE greater than this threshold are considered poor quality. In both cases, this threshold appears to separate the majority of good quality arrays from the long right tail of poor quality arrays.

To assess the overall quality of publicly available microarray data, we computed GNUSE values for all Affymetrix HGU133a and HGU133plus2 MIAME-compliant arrays available from the Gene Expression Omnibus (GEO) [22] in December, 2009. In total, we assessed 11,299 Affymetrix HGU133a microarrays from 338 experiments and 11,029 Affymetrix HGU133plus2 microarrays from 471 experiments for a total of 22,328 arrays from 809 studies. While most GNUSE values are close to one (indicating acceptable quality), the long right tails demonstrate that their are some probesets on some arrays that are of very poor quality (Figure 4). In fact, many of these poor quality probesets come from the same arrays (Figure 5).

Based on the MAQC data described above, we observed that removing arrays whose GNUSE median exceeded 1.25 improved prediction. One can interpret this threshold as filtering arrays whose precision is on average 25% worse than the typical array. Based on this threshold, roughly 12.1% of HGU133a arrays and 7.6% of HGU133plus2 arrays are of poor quality. The distribution of GNUSE medians along with this threshold further supports the GNUSE median threshold as providing reasonable separation between the majority of arrays with acceptable quality and those with poor quality (Figure 5).

#### Sources of Poor Quality

**Figure 6:**
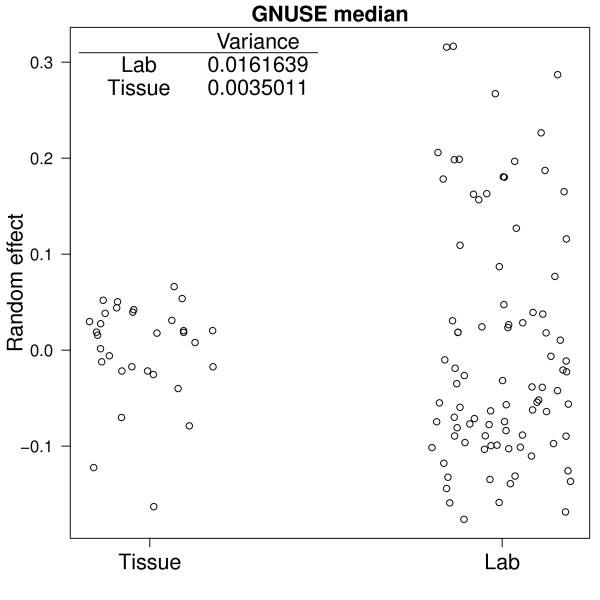
**Lab accounts for more variability in quality than tissue type**. Resulting from fitting Model 2. The individual random effects for lab and tissue are plotted and the estimated variance for each effect is reported. These estimates suggest that the lab in which an array was hybridized accounts for more of the variability in microarray data quality than the tissue that was hybridized to the array.

We now turn our attention to the potential causes of poor microarray quality. First, we examined 4,456 microarrays from 120 studies consisting of arrays publicly available through ArrayExpress [23] or GEO for which the lab in which the array was hybridized could be ascertained. We focused on two potential sources of poor microarray quality - the type of sample analyzed or the laboratory in which the sample was analyzed. To investigate these sources, we fit the following random effects ANOVA model:

with,

where *μ *is the overall average GNUSE median across all *i *samples, *j *sample types, and *k *labs. *S_j _*is the random effect for sample type *j*, *L_k _*is the random effect for lab *k*, and *ε_i,j,k _*represents measurement error. We can assess the variability in GNUSE medians by comparing the estimated variance of sample type effects, , and the estimated variance of lab effects, . The estimated variance between labs is more than 4 times greater than the estimated variance between sample types (0.0162 vs 0.0035), suggesting that the lab in which an array was hybridized accounts for more of the variability in microarray data quality than the tissue that was hybridized to the array. Figure 6 shows the individual lab and tissue effects as well as their estimated variances.

Furthermore, we fit a one-way ANOVA model of the GNUSE medians (log-transformed) on lab separately for two tissue types analyzed by many labs - bone marrow and brain. Table 2 shows that most lab effects within each tissue are statistically significant (p-value < 0.05) and practically significant, with estimated lab effects of up to 23%.

**Table 2:** ANOVA model effects across labs

Bone Marrow	Quality difference	Sample size
Lab 1	-0.046*	35
Lab 2	0.168*	112
Lab 3	0.175*	20
Lab 4	0.064*	27
Lab 5	-0.111*	14
Lab 6	-0.053*	12
Lab 7	0.044*	43
Lab 8	-0.145*	19
F-statistic p-value	<.001	

**Brain**	**Quality difference**	**Sample size**

Lab 1	-0.054*	201
Lab 2	0.016	24
Lab 3	0.029*	31
Lab 4	0.229*	42
Lab 5	-0.053*	199
Lab 6	-0.042*	82
F-statistic p-value	<.001	

ANOVA model effects for GNUSE median across labs, for two tissue types - bone marrow and brain. There is a statistically significant difference in quality between labs in both tissue types (F-test p-values < 0.001). A * denotes a statistically significant lab effect.

#### Poor Quality Studies

Finally, we report the overall quality of the 809 MIAME-compliant microarray studies available via GEO in December 2009. For each study, we report the number of arrays, the average GNUSE median, and the proportion of poor quality arrays (GNUSE median > 1.25) in Additional file 2. The first result of interest is that array quality does not appear to be correlated with study size (correlation coefficient = 0.055).

There are 87 studies that are composed of at least half poor quality arrays. Some of these extremely poor quality studies can be explained by further examination of the experimental design - for example, GSE2703, GSE6814, and GSE907 hybridized Macaca mulatta RNA to HGU133a arrays designed to measure human gene expression. However, this only explains a handful of these poor quality studies.

## Conclusions

We have described microarray quality in general and provided the mathematical formalism that permits us to quantify the quality of a microarray hybridization. Using this formalism, we have demonstrated how to assess quality based on the 3 most common microarray applications and used these applications to describe the strengths and weaknesses of the most common quality metrics used to assess Affymetrix GeneChip microarrays. Specifically, we found that the methods proposed by Bolstad et al. are often able to detect poor quality arrays while the methods proposed by Affymetrix are not. However, the methods of Bolstad et al. are inherently multi-array, so we propose a single-array modification of the NUSE metric, called the GNUSE. We show that the GNUSE metric differs substantially from the NUSE metric only when the experiment is composed primarily of poor quality arrays.

We then use the GNUSE quality metric to assess the quality of publicly available microarray data. We found that roughly 10% of publicly available Affymetrix HGU133a and HGU133plus2 arrays are of poor quality. We also found that these poor quality arrays are not evenly distributed among labs or studies - that is, some labs are more likely to provide poor quality arrays than others, and some studies are compromised of mostly poor quality arrays.

While the most likely cause of high GNUSE values is poor array quality, it is conceivable that a study using a non-standard hybridization protocol or investigating a particularly unusual tissue type might appear to have poor quality. An example of the latter situation is the hybridization of non-human RNA to human microarrays. A potential example of the former situation may be the data used to create the BioGPS webtools [11]. The 158 arrays used in the creation of these webtools (GSE1133) showed consistently high GNUSE values - 63.9% of the arrays had a median GNUSE above 1.25 and 96.8% of the arrays had a median GNUSE greater than 1. It is difficult to determine whether these arrays are of nearly uniformly poor quality or simply differ from typical arrays in some manner. Nevertheless, combining these arrays with arrays from any other experiment would certainly not be advisable.

The greatest strength of the GNUSE metric, the ability to assess the quality of a single array relative to overall microarray quality, is also its primary limitation - it requires a sizable number of arrays from different labs and different tissues to assess overall microarray quality. However, with the rapid increase in microarray experiments, this limitation is quickly diminishing, and the advantages of the GNUSE metric are growing. While there have been previous attempts at providing array quality metrics coupled with publicly available data sets [24,25] and at assessing the effect of quality on differential expression [26], these attempts used metrics that could only assess the quality of an array relative to other arrays in the batch or the quality of a batch of arrays relative to other batches of arrays. The incorporation of the GNUSE metric in such efforts would allow one to truly assess the quality of publicly available data.

The results presented here are based on the two most widely used Affymetrix microarray platforms. As more data becomes available on newer platforms, we look forward to implementing fRMA and the GNUSE on those platforms. We currently have a preliminary implementation of fRMA on the Human Exon ST 1.0 array. Based on 874 publicly available arrays, roughly 4.5% of arrays have a median GNUSE greater than the quality threshold of 1.25 (Additional file 1, Figure S7). This may indicate that newer arrays are of better quality or that the quality threshold needs to be reassessed when measuring exon-level rather than gene-level expression.

While the results presented here focus primarily on Affymetrix GeneChip microarrays, many of the ideas can be generalized to other platforms and manufacturers. Specifically, we recommend defining quality in a quantitative manner that focuses on the bottom-line results from common genomic applications.

Furthermore, assessing the quality of one sample in the context of the wealth of public data is a powerful technique for developing quality metrics in high-throughput studies. We believe that the ideas and formalism described here can form the basis for future quality assessments of other microarray platforms and even other genomic technologies.

The GNUSE algorithm is available as part of the frma R package on Bioconductor [27].

## Authors' contributions

MM helped design the study, carried out some of the analyses, and wrote the manuscript. PM helped design the study, carried out some of the analyses, and helped prepare the manuscript. ML organized and annotated the data. WH helped conceive the paper. RI conceived the study, carried out some of the analyses, and helped write and edit the manuscript. All authors read and approved the final manuscript.

## Supplementary Material

Additional file 1 **Supplementary Figures**. Figures S1-S7. Click here for file

Additional file 2 **GNUSE by Study**. Table containing the overall quality of the 809 MIAME-compliant microarray studies available via GEO in December 2009. For each study, we report the number of arrays, the average GNUSE median, and the proportion of poor quality arrays (GNUSE median > 1.25). Click here for file
