# Supplementary material for: Assessing affymetrix GeneChip microarray quality
Source: BMC Bioinformatics. 2011 May 7;12:137. doi: 10.1186/1471-2105-12-137 (PMC3097162; doi:10.1186/1471-2105-12-137)
Supplement: Additional file 1 — Supplementary Figures. Figures S1-S7. [file 1471-2105-12-137-S1.PDF]

Additional File 1

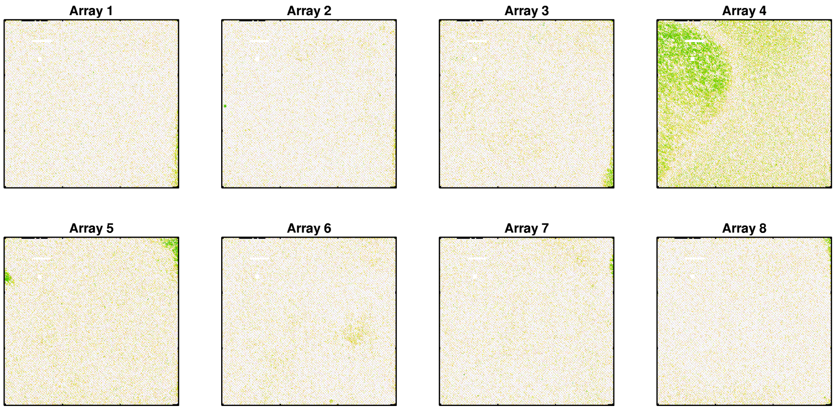

Figure S1 - Visual inspection clearly detects the poor quality array
Pseudo-images of the residuals from the 8 Affymetrix spike-in experiment arrays used to generate the ROC curves in Figure 1. While there appear to be small edge effects on many of the arrays, nearly all of array 4 is affected by a spatial artifact.

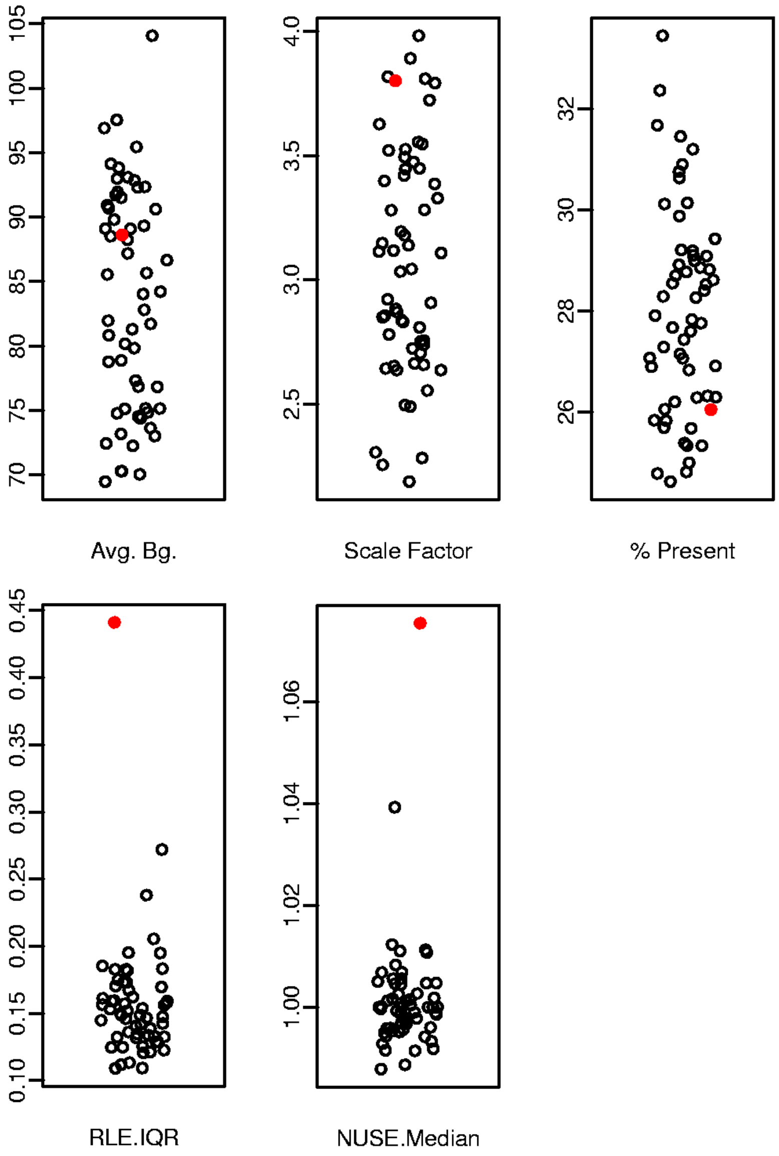

Figure S2 - Affymetrix quality metrics do not detect the poor quality array Quality metrics for the 59 arrays in the Affymetrix spike-in experiment. The poor quality array detected by the ROC curves in Figure 1 is shown in red. The top 3 quality metrics - average background, scale factor, and percent present – are those provided by Affymetrix. None of these are able to detect the poor quality array. However, the two quality metrics proposed by Bolstad et al. are able to detect the poor quality array.

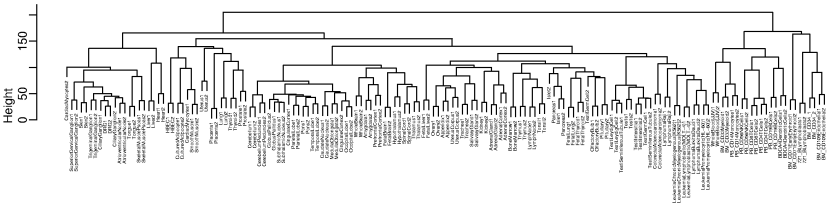

Figure S3 - Clustering of paired tissue samples Dendrogram displaying the hierarchical clustering of 79 different tissues on 158 arrays (2 arrays per tissue) based on Euclidean distance. Most tissue pairs clustered together; however, a few do not, suggesting one of them may be of poor quality. There is also a sub-cluster of tissue pairs at the far right that may be of suspect quality.

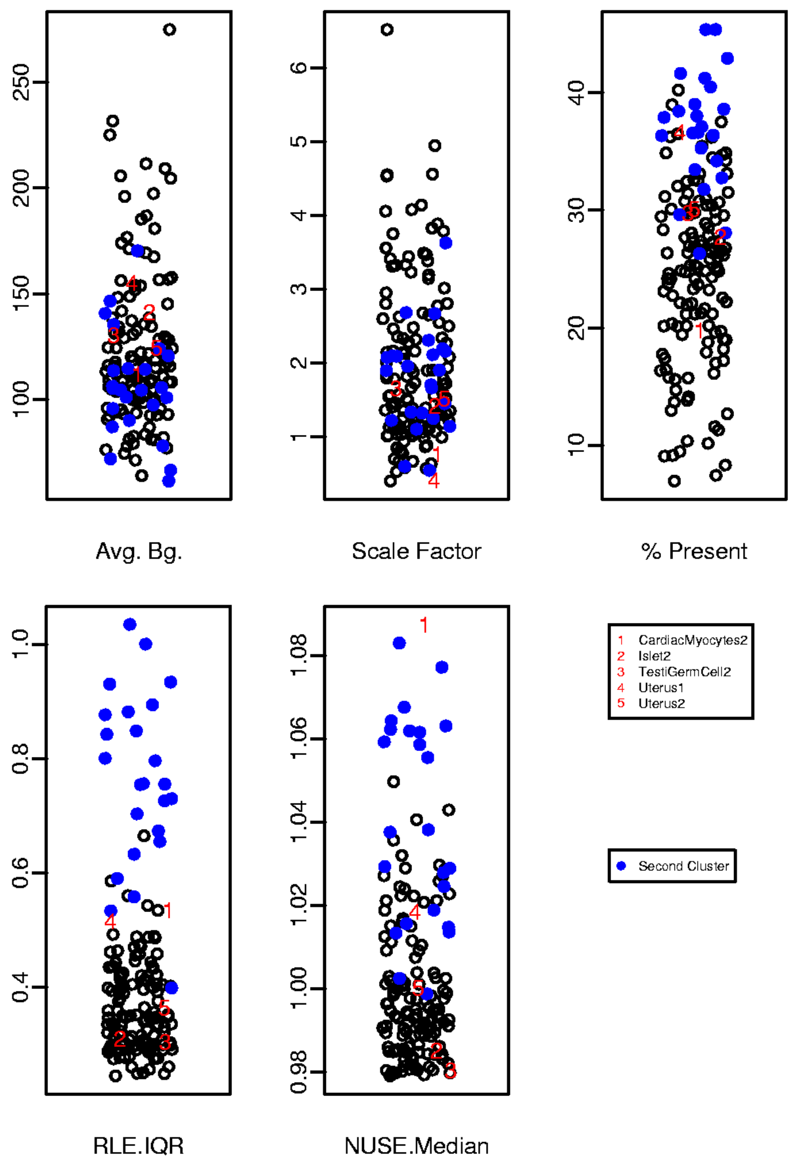

Figure S4 - Only the NUSE quality metric detects the poor quality array Quality metrics for the 158 arrays in the tissue pair data set. The poor quality Cardiac Myocytes array is denoted by a red 1. This array is only detected as poor quality by the NUSE quality metric. However, the sub-cluster identified in Supplementary Figure 3 is clearly detected as poor quality by the RLE metric and, to a lesser extent, by NUSE and present present.

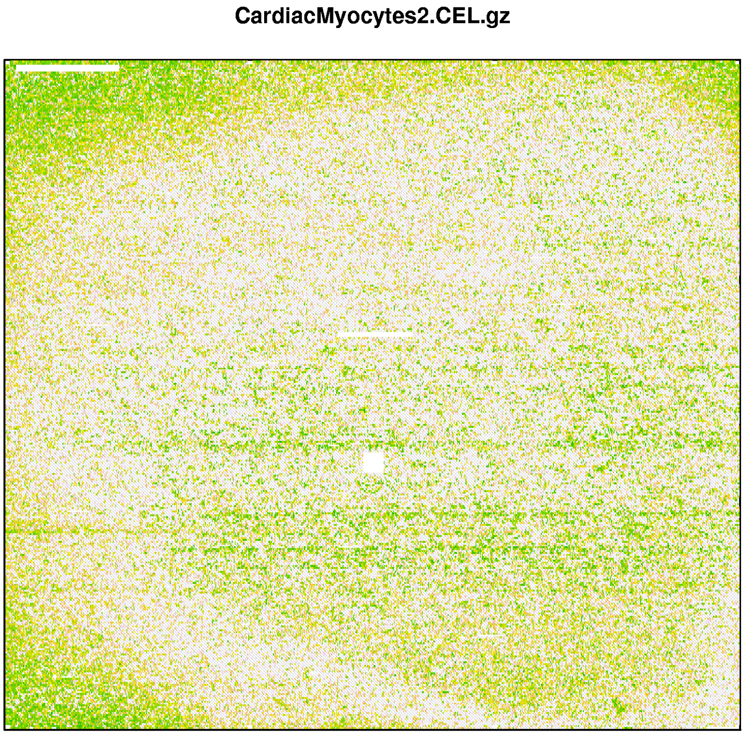

Figure S5 - Cardiac Myocytes pseudo-image Pseudo-image of residuals for the poor quality Cardiac Myocytes array detected by NUSE in Figure 6. There is a clear spatial artifact.

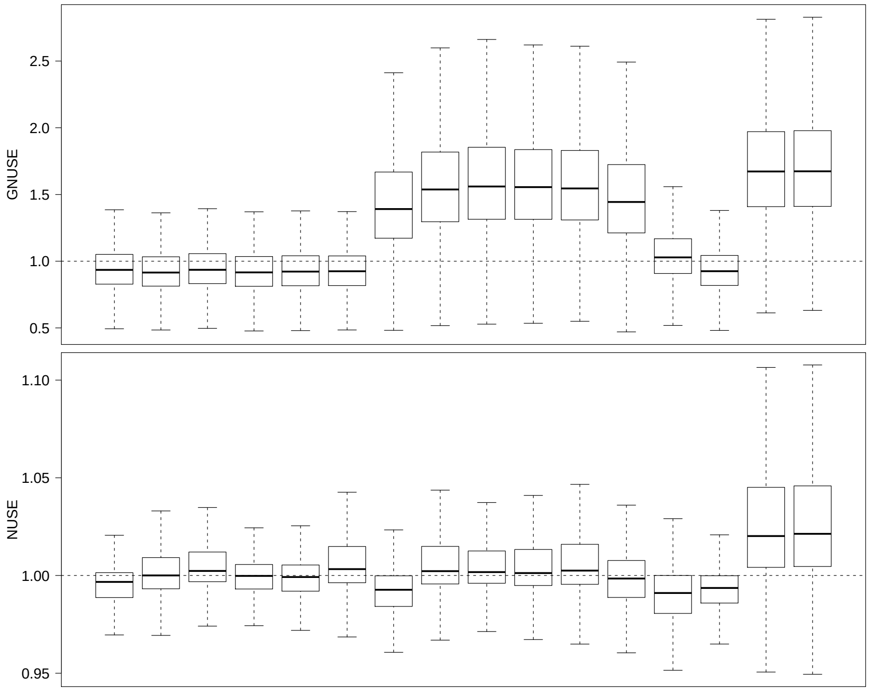

Figure S6 - GNUSE outperforms NUSE in studies with primarily poor quality arrays Boxplots of the GNUSE and NUSE values for a published study comprised of primarily poor quality arrays. The GNUSE is able to correctly assess the quality of arrays regardless of the overall quality of the study; the NUSE is unable to do so because it accepts the median array in the study as representative of acceptable quality.

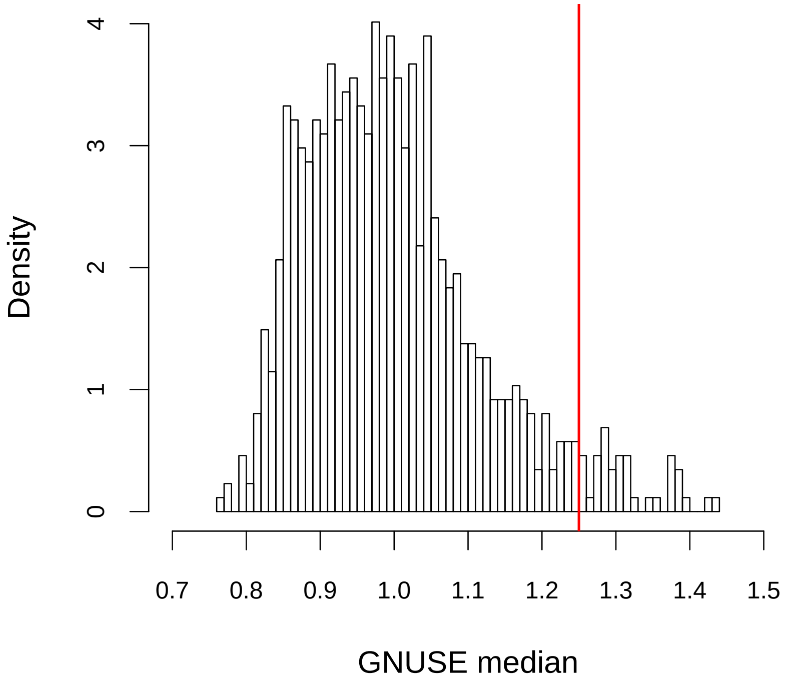

Figure S7 - Distribution of median GNUSE values for Human Exon arrays Histogram of median GNUSE values from 874 Affymetrix Human Exon ST 1.0 arrays. The red vertical line represents the threshold of 1.25 recommended for HGU133a and HGU133plus2 arrays – arrays with a median GNUSE greater than this threshold are considered poor quality. This threshold appears to separate the majority of good quality arrays from the right tail of poor quality arrays; however, for the Human Exon arrays the threshold might need to be slightly lower. Once more data is publicly available, this threshold will be reassessed.
